# Crimean-Congo Hemorrhagic Fever: An Emerging Viral Infection in India, Revisited and Lessons Learned

**DOI:** 10.7759/cureus.43315

**Published:** 2023-08-10

**Authors:** Aadil A Patel, Yagnya D Dalal, Amrita Parikh, Rajkamal Gandhi, Anand Shah

**Affiliations:** 1 Internal Medicine, Smt. NHL Municipal Medical College, Ahmedabad, IND; 2 Medicine, GCS Medical College, Ahmedabad, IND; 3 Medicine, Smt. NHL Municipal Medical College, Ahmedabad, IND; 4 Internal Medicine, Rutgers University New Jersey Medical School, New Jersey, USA

**Keywords:** hepatosplenomegaly, highly infectious, nosocomial spread, icterohaemorrhagic fever, cchf

## Abstract

Crimean-Congo hemorrhagic fever (CCHF) is a zoonotic disease caused by the CCHF virus. It was first recognized in 1944 in the Crimea region of the former Soviet Union and then was subsequently isolated in Congo, from a child with similar symptoms. Hence, the virus was termed the Crimean-Congo hemorrhagic fever virus. CCHF is an emerging disease with more than 1000 human cases being reported every year from South-Eastern Europe and Western Asia. The disease is endemic in Africa, the Balkans, the Middle East, and Asia, with an estimated 10,000 to 15,000 CCHF infections each year. The geographic range of the CCHF virus is most extensive among the tick-borne viruses that infect humans. The first outbreak of CCHF in India was described in 2011 in the state of Gujarat with four cases being reported. Since then, there have been sporadic cases in India occurring in small clusters with community and nosocomial spread. Here, we describe three cases that were treated at a tertiary care teaching hospital in the Gujarat state of India. All of them had nonspecific symptoms of viremia initially, followed by rapid deterioration of the general condition. Two of the three patients died. Because of its resemblance with other hemorrhagic fevers, diagnosis of CCHF remains a challenge, especially in non-endemic areas. We aim to sensitize the readers to this emerging arboviral disease because the virus is highly infectious and carries high mortality, and hence, it is crucial to suspect and diagnose the index case at the earliest.

## Introduction

Crimean-Congo hemorrhagic fever (CCHF) virus is a negative-stranded RNA virus included in the family Nairovirus. The virus has been found in about 30 different species of ticks. It is primarily transmitted via hard-bodied Hyalomma ticks of the family Ixodes. The disease is endemic in parts of Africa, south-eastern Europe, western Asia, and the Middle East [1-4]. The primary reservoirs of the virus are domestic livestock (cattle, sheep, pigs, and goats) infected by adult ticks. Birds spread the disease by carrying the CCHF-infected ticks over vast distances. Animals like rodents, hedgehogs, and the larvae and nymphs of ticks. Human transmission occurs due to a bite from an infected tick, crushing of an infected tick with the bare hand, direct contact with blood or bodily fluids of infected subjects, nosocomial transmission, and vertical transmission. Tick bites are reported by 60-69% of patients [5]. As the CCHF virus is highly infectious and easily transmissible; culture of this virus requires biosafety level four (BSL-4) [6].

Clinical features range from subclinical disease (88%) to viral hemorrhagic fever and multiorgan failure [7]. The mean incubation period is two to seven days. Symptoms during the pre-hemorrhagic phase are non-specific and can include sudden onset of fever, headache, myalgia, conjunctivitis, malaise, photophobia, nausea, vomiting, and abdominal pain [8,9]. Clinical findings can include lymphadenopathy and hepatosplenomegaly. The CCHF virus causes endothelial dysfunction causing capillary leakage of RBCs and plasma in the tissues [10], resulting in petechiae, ecchymosis, epistaxis, and bleeding from any site. Severe disease can result in cytokine syndrome causing hypotension, shock, multiple organ dysfunction syndrome (MODS), and death [9,11]. The convalescence period can last up to four weeks with no long-term sequelae. Laboratory manifestations include cytopenias, transaminitis, jaundice-elevated lactate dehydrogenase (LDH), prolongation of coagulation studies, and renal dysfunction. The diagnosis of CCHF is usually delayed during the non-specific symptoms phase due to lack of suspicion. Diagnosis is usually done using reverse transcription-polymerase chain reaction (RT-PCR). Specific IgM and IgG antibodies can also be used in resource-limited settings.

The first outbreak of CCHF in India occurred in the state of Gujarat in January 2011 with four cases [12]. The index patient was admitted with non-specific symptoms and rapidly progressed to hemorrhagic manifestations, multi-organ failure, and died on day 4 of admission.CCHF was not suspected in the index case and so was not tested. The patient was thought to be infected with the CCHF virus retrospectively [12]. The second and third cases were nosocomial contacts of the index case. Both tested positive for CCHF. Both of them also died. The fourth case was the husband of the index case. He was started on supportive therapy and Ribavirin after he developed nonspecific features of viral infection. He tested positive for CCHF but survived. Two of the three fatal cases had nosocomial transmission in the first outbreak. There was a re-occurrence of CCHF in Gujarat in June 2012. A shepherd presented with non-specific viral symptoms and progressed rapidly to multi-organ dysfunction with hemorrhagic manifestations. He died before CCHF was suspected or tested. A 29-year-old physician treating him had nosocomial transmission (splash of infected blood in his eyes). The physician’s blood sample tested positive for CCHF, and the treating physician died of CCHF. Based on these two outbreaks in the state, we formulated working criteria for suspecting unusual viral icterohemorrhagic fever consisting of the following parameters: geographical setting, exposure to cattle, occupational exposure, exposure to unusual viral hemorrhagic fever; negative serology for the common causes of hemorrhagic fevers in the area (dengue hemorrhagic fever, complicated malaria, leptospira); early, rapid, and severe thrombocytopenia, rapid evolution into disseminated intravascular coagulation (DIC), and early severe transaminitis. In 2019, 34 cases of CCHF were recorded in Gujarat with a case fatality of 50%. Here, we describe three cases that were admitted to our hospital.

## Case presentation

Case 1

A 75-year-old female cattle handler came with the chief complaints of intermittent high-grade fever with chills, abdominal pain, anorexia, nausea, and vomiting for four days. She had myalgia, generalized weakness, and yellowish discoloration of urine for two days. On presentation, the patient was febrile and hemodynamically stable. Examination revealed petechial rashes over the abdomen with hepatosplenomegaly. She had severe thrombocytopenia with severe transaminitis, leukopenia, and an altered coagulation profile. She had negative rapid antigen and serological tests for dengue fever and a negative smear for malaria. Her laboratory work is mentioned in Table 1. She developed severe breathlessness with hemodynamic instability within a few hours of admission. She was put on a mechanical ventilator and inotrope support. In view of the high suspicion of unusual viral hemorrhagic fever, along with her laboratory reports, clinical picture similar to cases of CCHF previously recorded, and relevant epidemiological risk factors. The sample was sent for CCHF viral PCR to the National Institute of Virology (NIV), Pune. She succumbed on the fifth day of illness on the day of hospital admission. Her CCHF RT-PCR was positive.

**Table 1:** Laboratory parameters and outcomes of the patients gm - grams, dl - decilitre, kU - kilo units, L - liter, U - units, mg - milligrams, sec - seconds, SGPT - serum glutamic pyruvic transaminase, SGOT - serum glutamic oxaloacetic transaminase

Investigations	Normal Range	Case 1	Case 2			Case 3		
		Day 1	Day 1	Day 5	Day 11	Day 1	Day 5	Day 11
Haemoglobin gm/dl	12-18	11.8	15	9.7	9.6	12.6	8.6	8.7
WBC kU/L	5.2-12.4	2.65	1.4	2.69	22.25	2.57	6.05	5.7
Platelet kU/L	130-400	8	33	16.4	2.68	22	56	467
SGPT U/L	10 - 49	1402	49	48		111	99	66
SGOT U/L	0 - 34	5413	120	69		307	245	
Creatinine mg/dl	0.5-1.3	1.64	1.39	1.03	3.8	0.76	0.53	0.57
Urea mg/dl	9-23	100.6	79.2	102.7	257	21.4	20.2	
Bilirubin mg/dl Total/Direct	0.3-1.2 / 0-0.2	2.97/2.45	5.3/0.78	2.53/1.21		0.8/0.4	1.22/0.42	1.63/0.6
Prothrombin time (sec)/INR	12-16 / 0.8-1.2	22 /1.88	20.4 /1.69			12.8 /0.93	12.8 /0.94	
Outcome		Expired			Expired			Discharged

After the identification of the index case, extensive contact tracing of households, neighborhoods, abattoir workers, and healthcare workers was done and 33 more cases of CCHF were identified.

Case 2

A 20-year-old male laborer from rural Gujarat presented with high-grade fever with chills, headache, myalgia, and vomiting for four days. He had decreased responsiveness and disorientation since one day. The patient’s relatives gave a history of contact with a co-worker who had died of unusual viral icterohemorrhagic fever. On presentation, the patient was febrile with tachycardia, tachypnea, and normal blood pressure with altered sensorium without neck rigidity and focal neurological deficit. General examination revealed petechial rashes all over the body along with icterus. He had significant thrombocytopenia and a mildly altered coagulation profile. He had negative antigen and serological tests for dengue fever and leptospira and the smear was negative for malaria. CCHF was suspected on epidemiological grounds and CCHF RT-PCR was sent to NIV Pune, which came positive. His laboratory reports are mentioned in Table 1. His blood sample was positive for CCHF. The patient was treated with ribavirin and supportive treatment. His neurological symptoms improved. On the eighth day of illness, the patient’s respiratory condition worsened. In view of decreasing saturation and worsening acute respiratory distress syndrome (ARDS), he was put on a mechanical ventilator and inotropic support. His condition and laboratory parameters continued to worsen. He died on the eleventh day of illness.

Case 3

A 34-year-old male working at a dairy farm came to our hospital with complaints of one episode of low-grade fever, body aches, and headache for six days, for which he took over-the-counter drugs. The fever resolved and subsequently, he developed hematuria and oral and nasal bleeding. The patient was admitted to a private hospital for two days where he was given platelet transfusion. He gave a history of a recent tick bite. On examination, the patient was vitally stable. There was no active bleeding from any site. He had severe thrombocytopenia with mild transaminitis and leukopenia. He had negative rapid antigen and serological tests for dengue fever and a negative smear for malaria. His laboratory investigations are mentioned in Table 1. His RT-PCR and immunoglobulin M (IgM) were positive for CCHF. The patient received supportive treatment and ribavirin. He improved and was discharged on the eleventh day of admission.

## Discussion

The incidence of CCHF is on the rise. Possible causes of outbreaks in previously disease-free areas include climate change [13], increased movement of domestic animals, changing agricultural practices, migrating birds, and increasing tick population [1]. The case fatality rate of CCHF is 3-30% [13]. The outbreak in Gujarat in 2019 had a mortality rate of more than 50%. Endemic areas with large disease incidence have low case fatality rates. The reason behind this is not known.

Nosocomial transmission of CCHF is common and has been described in the literature. A review of the published nosocomial infections of CCHF in endemic and non-endemic countries reported a case fatality rate of 32.4%, with most (92.4%) of the cases being symptomatic [14]. The majority of nosocomial infections have been reported among healthcare workers. The mortality rate for nosocomially acquired CCHF infections is much higher than that acquired through tick bites, however, the reason for this is not known [15]. The spread of CCHF in a nosocomial setting is due to contact of infected body fluids with mucosa, skin, or percutaneous tissue. In a non-endemic country like India, nosocomial transmission is highly possible especially before an index case is suspected and tested.

Clinical features of CCHF in the pre-hemorrhagic phase are similar to any other viral infection and include fever, chills, rigors, myalgia, and gastrointestinal symptoms. The clinical picture, especially in the early stage of the disease, often overlaps with common endemic diseases like malaria, dengue, and leptospirosis. However, CCHF can be differentiated from these on the basis of early and rapid worsening of coagulation profile, early severe thrombocytopenia, early severe transaminitis, rapid rhabdomyolysis, and appropriate epidemiological history.

Treatment of CCHF includes isolation of the patient to prevent nosocomial spread. Supportive treatment with adequate hydration and the monitoring of renal function and fluid status should be done. Patients with altered coagulation profiles and/or bleeding should receive blood products. Ribavirin is the only specific antiviral treatment used in patients with CCHF. However, the evidence available for efficacy is only based on observational studies, and strong evidence is lacking. The effectiveness of ribavirin is not well-established and remains controversial. Some studies suggest mortality benefits when given early in the disease course [13]. Therefore, early diagnosis and initiation of ribavirin therapy should be done. Relevant epidemiological history like occupation, travel to endemic regions, history of tick bite, and meat handling should always be taken. In the case series described here, two of the three patients received ribavirin and one survived.

## Conclusions

It is of utmost importance to keep a high index of suspicion for any unusual viral icterohemorrhagic fever, particularly in a country where CCHF is sporadic. There is a need for predictive scoring systems to suspect various viral hemorrhagic fevers based on local epidemiological and clinical features. Rapid diagnosis of the index case can help in preventing the disease's spread in the community and among healthcare workers and is crucial to undertaking relevant epidemiological surveys to detect the source and mode of importation in the areas of outbreak. Ribavirin therapy may be helpful and should be started early in suspected cases. Implementation and periodic checking for adherence to standard and special precautions for undiagnosed hemorrhagic fever patients are essential to prevent the nosocomial transmission of CCHF.
